# Impact of BMGIM Music Therapy on Emotional State in Patients with Inflammatory Bowel Disease: A Randomized Controlled Trial

**DOI:** 10.3390/jcm10081591

**Published:** 2021-04-09

**Authors:** Vicente Alejandro March-Luján, Vicente Prado-Gascó, José María Huguet, Xavier Cortés, José María Paredes Arquiola, María Capilla-Igual, María Josefa-Rodríguez-Morales, Ana Monzó-Gallego, José Luis Platero Armero, José Enrique de la Rubia Ortí

**Affiliations:** 1 Department of Teaching and Learning of Physical, Plastic and Musical Education, Catholic University San Vicente Martir, 46110 Valencia, Spain; 2 Department of Social Psychology, University of Valencia, 46010 Valencia, Spain; vicente.prado@uv.es; 3 Digestive Disease Department, General University Hospital of Valencia, 46014 Valencia, Spain; josemahuguet@hotmail.com (J.M.H.); anamonzo2002@hotmail.com (A.M.-G.); 4 Digestive Disease Department, Hospital of Sagunto, 46520 Valencia, Spain; xacori@gmail.com (X.C.); prmorales06@yahoo.es (M.J.-R.-M.); 5 Universidad Cardenal Herrera-CEU, CEU Universities, 46113 Valencia, Spain; 6 Digestive Disease Department, Peset University Hospital of Valencia, 46017 Valencia, Spain; chemaparedes1969@gmail.com; 7 Research Foundation of the General Hospital of Valencia, 46014 Valencia, Spain; capigumar@gmail.com; 8 Department of Nursing, Catholic University San Vicente Martir, 46001 Valencia, Spain; jlplatero@hospitalmanises.es (J.L.P.A.); joseenrique.delarubi@ucv.es (J.E.d.l.R.O.)

**Keywords:** inflammatory bowel disease, music therapy, guided imagery, emotional aspects, anxiety, depression

## Abstract

Background. Patients with inflammatory bowel disease (IBD) have a high prevalence of emotional disturbances which worsen the symptoms of the disease. As a therapeutic alternative that is part of a comprehensive care alongside medication, the Bonny Method of Guided Imagery and Music (BMGIM) music-assisted therapy has achieved promising emotional improvements in patients with chronic diseases. The objective of the study was to determine the impact of a treatment based on a BMGIM group adaptation on patients with inflammatory bowel disease (IBD) and their emotional state, therefore analyzing state of mind, quality of life, anxiety, depression, immunocompetence as a marker of well-being, and levels of acute and chronic stress. Methods. Longitudinal, prospective, quantitative, and experimental study including 43 patients with IBD divided into an intervention group (22 patients), who received eight sessions over eight weeks, and a control group (21 patients). A saliva sample was taken from each patient before and after each session in order to determine cortisol and IgA levels. Similarly, a hair sample was taken before the first and after the last session to determine the cumulative cortisol level. All molecules were quantified using the ELISA immunoassay technique. In addition, patients completed several emotional state questionnaires: HADS, MOOD, and CCVEII. Results. An improvement was observed in the following states of mind: sadness, fear, anger, and depression. No significant effect was observed in state of mind in terms of happiness or anxiety, in the levels of cortisol in hair, and in patients’ perceived quality of life. A reduction in cortisol was observed in saliva, although this did not significantly affect the IgA titer. Conclusions. BMGIM seems to improve the emotional state of patients with IBD.

## 1. Introduction

Inflammatory bowel disease (IBD) includes Crohn’s disease (CD) and ulcerative colitis (UC). It is characterized by its chronic course, as well as by a fluctuating and recurrent clinical course [1] that impacts on mental health [2]. In this sense, these patients have psychological manifestations [3,4,5,6,7,8,9]. IBD affects patients comprehensively across multiple health domains, including diminished quality of life [10,11,12,13], depression and anxiety [14,15], low mood [16], and diminished well-being with a greater prevalence of emotional stress [17,18]. Diminished quality of life in patients with IBD is directly correlated with anxiety and depression [19], both of which are linked to and highly prevalent in chronic diseases [20]. Anxiety and depression, as well as alterations in psychological well-being, are positively correlated with inflammation and physiological stress markers in IBD [21,22] that are directly related to the immunocompetence of the individual. As a consequence, the evaluation of an ill person’s perception of well-being is closely related to immunocompetence. In this sense, immunoglobulin A (IgA) is a key part of the immune system that acts against threats, especially in autoimmune diseases such as IBD. This immunoglobulin is positively correlated with the well-being of the patient [23].

IBD patients also have high levels of emotional stress, related to the clinical manifestations of the disease [24,25,26] and characterized by increased secretion of the hormone cortisol [27].

As for therapeutic options, the emotional aspects of the disease are making treatments aimed at improving patients’ quality of life increasingly important [11,28]. In this sense, current nonpharmacological therapies are proving effective as a supplement to drugs [29,30,31]. Such is the case of cognitive-behavioral psychological treatment [3,4,32], mindfulness [33,34,35,36,37], guided imagery, and relaxation training [38]. Music therapy has also proven effective in treating different chronic diseases and their effects [39,40,41,42,43,44,45,46]. Music therapy comprises various models and methods [47,48]. A key method is the Bonny Method of Guided Imagery and Music (BMGIM) [49]. BMGIM is a working method employed by the transpersonal therapy institute, which uses edited music to explore the psyche by means of nontypical states of consciousness [50], achieving improvements in emotional and cortisol levels in patients with various pathologies [51,52,53,54,55,56,57], especially in group adaptations [58,59,60,61].

The main objective of this study was to determine the impact of a BMGIM group adaptation on the emotional state of IBD patients by analyzing changes in quality of life, perceived anxiety, depression, as well as in the mental state, i.e., sadness, fear, anger, and happiness. This also included the levels of IgA in saliva and cortisol in saliva and hair, as markers of well-being and emotional stress, respectively.

## 2. Materials and Methods

A randomized controlled trial (RCT) was conducted by means of a clinical trial. The clinical trial ID for this study is NCT03999294.

### 2.1. Participants

**Figure 1:**
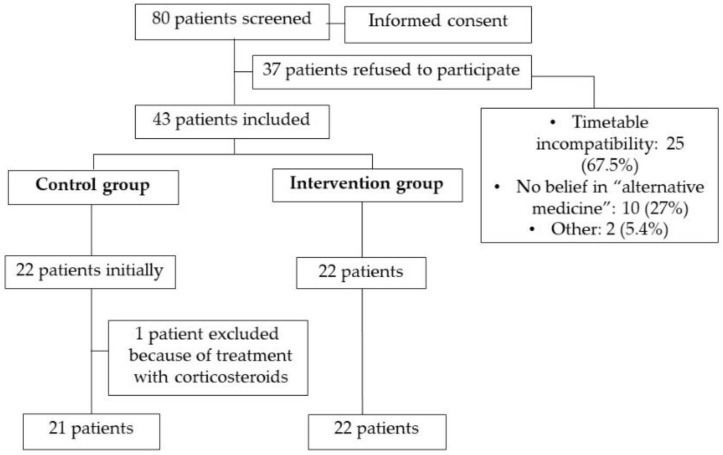
Flow chart.

The candidates that participated in the study were adults (over 18 years of age) diagnosed with IBD (CD and UC) in the remission phase, who visited the digestive pathology service at the Hospital General de Valencia and who were receiving the usual treatments for this disease. Furthermore, in order to obtain the desired sample size, patients from the same pathology service of the Hospital de Sagunto, also located in Valencia, were contacted. In order to calculate the sample size from the preliminary data of a pilot study carried out by our group, the minimum expected change in any of the dimensions of the MOOD scale was obtained (chosen because it is the necessary tool to address the main objective of the study, evaluating the emotional improvements of the intervention), which was 0.3, with an expected variance of change equal to 0.35. In order to detect this change, with a power of 80% and a confidence of 95%, it would take 46 patients (23 in each group). In order to obtain this population, all patients from the two hospitals (Hospital General de Valencia and Hospital de Sagunto) were previously informed of the characteristics of the study through an information sheet. Patients who were willing to participate in the study signed an informed consent prior to applying the selection criteria (Figure 1). Selection criteria were finally applied to 80 patients. The inclusion criteria were as follows: 1. Patients had to be aged ≥18 years and with CD or UC in remission. Remission was defined as a partial Mayo index < 2 points for UC and a Harvey–Bradshaw index < 5 points for CD. 2. No corticosteroids for at least 2 months before the initiation of the study. 3. Signature of the informed consent document. The exclusion criteria were as follows: 1. Refusal to participate in the study owing to aversion to or rejection of the therapy. 2. Taking corticosteroids for the 8 weeks in the therapy phase. A simple randomization was used to allocate the participants into the control or the intervention group. The researcher who enrolled the participants was different from the person who allocated the participants to the interventions. After allocating the subjects to the interventions, the researchers assessing the outcomes were blinded.

### 2.2. Ethical Concerns

Written informed consent was obtained from all participants before the study began. The study was approved by the Ethics Committee of Hospital General Universitario de Valencia and conducted according to the basic principles of biomedical research set out in the Declaration of Helsinki [62].

### 2.3. Procedure

This was a longitudinal, prospective, quantitative, and experimental study. Participants received information on the study including the defined objectives and the tests and analyses to be carried out and signed an informed consent. Before the intervention, they were also provided with instructions not to change their lifestyle and not to participate in any other type of therapy or activities that could interfere with the variables to be analyzed. Patients were managed according to the physician’s criteria, with no restrictions on treatment for IBD.

### 2.4. Intervention

Once the selection criteria had been applied, a final sample of 43 patients was obtained, and the participants were randomly allocated to the intervention and the control groups. A randomization process without stratification was carried out by selecting sealed, opaque envelopes that had been previously arranged in a computer-generated random order. Once the population had been divided into the two groups, the study was carried out between April 2016 and January 2017 in the inflammatory bowel disease departments of the Hospital General Universitario de Valencia and Hospital de Sagunto (Spain). Patients visiting the clinic during the recruitment period were randomly offered to participate in the study. After their medical appointments, a trained member of the research team explained to the patients that participation in the study was completely voluntary and anonymous and that a written informed consent form would need to be signed.

Sessions were conducted by a postgraduate professional qualified in Music Therapy (Universidad de Barcelona) and a specialist in BMGIM Music Therapy method (Atlantis Institute (USA)). BMGIM is one of the approaches currently recognized by the international music therapy community and must be conducted by a qualified professional (Master’s degree in Music Therapy or Bachelor’s degree depending on the standards of professional development and training in Music Therapy in each country, as well as having completed a specific training in this method). The intervention group received 8 BMGIM sessions adapted to small groups (5–6 patients per group) over the 8 weeks of the duration of the intervention on the same day of the week (Tuesday) and at the same time (9:00 a.m.), each one lasting 2 h. These groups were divided in this way by taking into account the optimal results of other studies that used similar distributions and that had also used a group adaptation of BMGIM [60,61]. The structure of each of the sessions followed a similar sequence as that originally proposed by Grocke and Wigram [63] and later replicated in a recent study by Torres, Pedersen and Pérez-Fernández [60]. In this sense, and in accordance with what these authors indicate, the dynamics of each one of the sessions was constant and consisted of the following phases/stages, always in the same temporal order:

- Initial verbal phase (oral stage);
- Induction;
- Music listening phase/stage;
- Return to ordinary awareness and creative integration of the experience;
- Final verbal phase/stage sharing.

On the other hand, the control group did not receive any sessions of any kind, remaining in a room in the same premises, while the intervention group sessions took place.

Half an hour before each session (8:30 a.m.), a saliva sample was taken from each patient, and the questionnaires were completed (the questionnaires were filled in by the patients themselves in the waiting area in 30 min and then given to a member of the research team). The process was repeated after each session (11:00 a.m.). A hair sample was also taken from each patient before the first and after the last session. Both saliva and hair samples were taken from the control group at the same times. The control patients also filled in the same questionnaires as the intervention group over the 8 weeks of the intervention.

### 2.5. Measurements

The quality of life of patients with IBD was evaluated using the Short-form Questionnaire on Quality of Life in IBD (CCVEII-9) [64], which is a short version of the Spanish version of the 32-item Inflammatory Bowel Disease Questionnaire (IBDQ). The final score is expressed on a scale of 0 to 100 points, such that a lower score corresponds to a lower quality of life. The Cronbach alpha for this scale ranges from 0.90 to 0.91.

In order to evaluate changes in the patient’s subjective perception for the variables of anxiety and depression, we used the Spanish version of the Hospital Anxiety and Depression Scale (HADS) [65,66], which comprises 7 items for each dimension and has a Cronbach alpha ranging from 0.80 and 0.86 for both dimensions of the scale and around 0.90 for the total scale. The score for the items is evaluated on a Likert scale ranging from 0 to 3 points, where 0 represents never or almost never, 1 a little or from time to time, 2 somewhat less than before or not as intense, and 3 maximum or almost always. Lastly, it is worth mentioning that these scales are interpreted based on the sum of each dimension, with 21 as the maximum, both for depression and for anxiety.

The MOOD questionnaire [67] was used in its adapted format validated in Spanish by Górriz, Prado-Gascó, Villanueva, Ordóñez and González [68]. The scale evaluates the frequency of the different states of mind (happiness, anger, sadness, and fear) based on 20 items (4 dimensions of 5 items each). The responses for the items were measured using a 3-point Likert scale (1 = never, 2 = sometimes, 3 = often). The internal consistency of the Spanish version of this scale was good, with a Cronbach alpha ranging from 0.69 to 0.78 for the 4 dimensions (sadness α = 0.69; fear α = 0.69; anger α = 0.78, and happiness α = 0.76) and 0.85 for the total scale.

Physiological well-being and stress level were assessed by quantifying the biological markers IgA and cortisol, in saliva and hair samples using the enzyme-linked immunosorbent assay (ELISA). IgA is found in several types of secretion and is measured mainly in saliva [69]. In order to determine emotional stress at the physiological level, the measurement of cortisol provides objective and effective information [27], thus making it the most widely determined marker in various diseases [70,71,72]. Acute stress in particular is usually assessed by measuring the hormone in saliva, where values are well categorized [72]. However, the level of stress in the medium term, is better quantified based on the level of cortisol in other types of samples, mainly hair, where secretion is more stable during the months immediately preceding sampling [73]. In the case of the saliva samples, at least 2 mL of unstimulated saliva was collected in 10 mL sterile plastic tubes that were then stored in a container with ice. The procedure had an approximate total duration of 5 min. The samples were then centrifuged in the laboratory at 1500× *g* for 15 min, and the supernatant was frozen and stored in microsample tubes at −20 °C until the samples were ready to be analyzed. To determine the IgA level, the Salivary IgA ELISA SLV-4636 kit protocol (R&D Systems, Minneapolis, MN, USA) [74] was followed. In the case of cortisol, we used the protocol of the Salivary Cortisol ELISA SLV-2930 kit (DRG International Inc., Springfield, NJ, USA). The hair samples were cut from the base of the neck with fine scissors, taking approximately 100 strands of hair of at least 3 cm in length for each patient. Each sample was then pulverized with the scissors to facilitate the extraction of cortisol, before being incubated with 1 mL of methanol at 34 °C and shaken gently for 36 h. The supernatant was then separated and evaporated in an evaporator connected to a flow of nitrogen in order to improve the process. The plaques were placed on a hot plate to further favor evaporation. The resulting pellet was redissolved with phosphate-buffered saline. The salivary cortisol level was then measured with the SLV-2930 kit (DRG International Inc.) [73].

### 2.6. Statistical Analysis

The statistical analysis involved the calculation of the main descriptive parameters (median, mean, range, standard deviation). Analysis of covariance (ANCOVA) was then performed to determine the effect of treatment on the study variables. The pretest scores were used as covariates, the type of treatment as between-subject factor, and the post-test scores as the dependent variables. The analyses were performed using IBM SPSS, Version 24 (IBM Corp, Armonk, NY, USA, 2016). A chi-square test was used to analyze categorical data. A *p*-value below 0.05 was considered significant.

The pre-ANCOVA assumptions were verified with 3 assumptions: (1) that the covariate variable had a statistically significant effect on the post-test treatment score; (2) the nonexistence of a statistically significant effect between the covariate variable and the independent variable treatment; and (3) the homogeneity of the regression slopes. In this way, the covariate variable had a statistically significant effect on the post-test treatment scores (*p* < 0.05 in all cases). In addition, the nonexistence of a statistically significant effect between the covariate variable and the independent variable treatment was confirmed (*p* > 0.05 in all cases). Moreover, the homogeneity of the regression slopes or the absence of a statistically significant interaction between the covariate variable and the variable treatment was confirmed (*p* > 0.05 in all cases). Therefore, it seems that all of the previous assumptions for performance of the ANCOVA were fulfilled in all cases. The ANCOVA was performed between single-factor groups with 2 conditions: nontreatment group and music therapy group for each of the study variables. The covariate variable was the pretest score for both groups.

## 3. Results

### 3.1. Baseline Characteristics of the Study Group

Of the 80 patients originally interested in the study, 37 patients finally refused to participate (as shown in Figure 1). The remaining 43 patients were redistributed, following the randomization criteria: 22 were included in the intervention group, and 21 in the control group. One patient was excluded from the control group for taking corticosteroids during the study; therefore, the final number of patients was 21 (see Figure 1 for the patient flow chart).

The baseline characteristics of the final study population are shown in Table 1. When comparing these variables between groups (chi-square test), no significant differences were observed, except for the variable without treatment (*p* < 0.026) in the case of patients without medication in the intervention group, and for the variable with anti-TNF treatment (*p* < 0.13) in the case of the control group, with were more patients who took these drugs.

### 3.2. Measurement of Emotional Variables

Table 2 shows the mean and standard deviation and the main results of the ANCOVA for the dimensions of the questionnaires MOOD, CCVEII, and HADS and for the biological markers of the hormone cortisol (in saliva and hair) and IgA (in saliva), taking into account the pre-test and post-test values in both the control and the intervention group.

When the post-treatment results were compared between the groups after controlling the pretreatment scores (Table 2), a reduction in the levels of the states of mind of sadness, fear, anger, depression was observed; no change was found for the states of happiness, anxiety, or perceived quality of life.

As for the results obtained for the biomarkers, determined in both saliva and hair, a significant decrease in salivary cortisol concentrations was observed; however, there were no changes in the levels of cortisol in hair or of salivary IgA, and the differences that were observed were not statistically significant.

## 4. Discussion

The results obtained in our study after the intervention based on a BMGIM group adaptation in patients with IBD show significant improvements in most of the psychopathologic variables analyzed, as well as in acute physiological stress levels determined from cortisol levels in saliva. Therapies that are part of comprehensive care alongside pharmacological–medical therapies, such as music therapy, are currently achieving optimal outcomes in patients with chronic inflammatory diseases (in terms of quality of life, state of mind, and stress level) [75,76]. The BMGIM approach in particular has proven to be very successful in reducing stress in patients with various diseases [54,58,60].

The analysis of the impact of BMGIM on perceived quality of life did not show significant changes in the intervention group. When these findings are compared with those in the literature, the results are contradictory, since studies, such as those by Burns [77], Bonde [78], and Maack [52], did show a significant increase in perceived quality of life in patients who received BMGIM. However, the duration of these studies was different (Burns’s study [77] lasted 10 weeks, Bonde’s [78] 20 weeks, and that of Maack [52] varied in duration depending on the type of patient), and fundamentally the nature of the diseases was also completely different, as in these studies only women with posttraumatic stress disorder or cancer patients, were treated. Therefore, it can be concluded that improvements in perceived quality of life depend mainly on the nature of the pathology or disorder. As for IBD patients, we infer it is necessary to carry out more studies in which the number of sessions, application time, or intensity are increased, trying to establish which is the protocol that can achieve significant improvements in this variable or, on the contrary, confirm that BMGIM does not improve quality of life in these patients. As for the psychopathological aspects of anxiety and depression in patients with IBD [6,15,79], after eight sessions, anxiety diminished but not significantly. In this sense, previous studies do report a significant reduction in perceived anxiety and in the state of anxiety after application of several BMGIM sessions [51,58,60]. Moreover, this observation has also been reported for other diseases, since anxiety is diminished significantly after applying several BMGIM interventions [54,56,57]. However, conversely, it was observed that depression as perceived by patients with IBD diminished significantly at the end of the BMGIM intervention; these results seem to be in line with those of other studies that analyzed the presence of symptoms of major depression and found a significant decrease after several BMGIM sessions [54,56,57]. Thus, the results obtained in this study do not reinforce the hypothesis that changes in anxiety are related to changes in depression, as indicated by other authors [75,79]. As for patients’ state of mind, a significant improvement was observed in sadness, fear, and anger, but not in happiness. These results are similar to those of other studies based on BMGIM, which reported a statistically significant improvement in the general state of mind after several sessions [54,55]. No previous studies specifically examined the variable happiness, thus precluding a comparison of the results with respect to this state of mind. After the analysis of all these variables, it would also have been interesting to examine if the emotional improvements were maintained over time, after stopping BMGIM. This aspect was not taken into account in this study. In this sense, in the current literature there are not many articles that have taken into account this factor, although the work of Torres et al., in 2018, should be highlighted. In this study, the researchers observed that the improvements in anxiety or depression were maintained for at least 3 months for patients with fibromyalgia [60].

Furthermore, the influence of therapy was assessed at the physiological level. The results of this study showed that IgA secretions decreased, although not significantly. It was also noteworthy that studies based on BMGIM reported similar findings. Heiderscheidt [80,81] obtained similar results, that is, there was no significant change in IgA levels after BMGIM in patients in the intervention group with respect to the control group. These results can be explained by the values obtained for happiness, which, in turn, is the only variable for which no satisfactory changes were observed after the intervention. In this sense, it is worth remembering that the association between perceived happiness and IgA titer has been known for some time [82,83], thus explaining why neither of the two variables improved, since they appear to be related. In addition, our results are also consistent with those of other studies in which the authors, despite not investigating music therapy, analyzed how singing and listening to music affect IgA in those who sing and those who listen, respectively. IgA increased in those who played music [84], but not so much in those who listened passively [85].

Finally, in terms of perceived stress, the results showed a significant decrease in the intervention group, although the difference was not significant after therapy. These results should be analyzed in greater depth. Therapy clearly improved stress in the medium term (based on hair cortisol concentrations), since the difference, as stated, was significant. However, this was not seen as differences between the populations, possibly because of readjustments in the hypothalamic–pituitary–adrenal (HPA) axis. In this sense, it is noteworthy that baseline cortisol values in saliva were very different (control group patients had much higher levels [9.74 ηg/mL] than intervention group patients [5.34 ηg/mL]), thus leading to negative feedback [86], as seen in Alzheimer’s disease [75] and as can be seen in hair cortisol levels in the long term. This feedback process is not observed in other types of samples, such as blood or even saliva, because at acute levels, the so-called negative feedback disinhibition process occurs [87], so that cortisol levels in this type of sample do not decrease, thus accounting for the absence of differences in hair between the groups. When the results of this study were compared with those obtained in other studies, no authors measured the long-term impact of BMGIM on stress levels in this type of patients using hair cortisol. Therefore, this study is the first to address this issue.

It is noteworthy that a significant reduction in acute stress was observed, as seen by the secretion of cortisol in saliva, thus indicating that the therapy does in fact lead to an immediate change in physiological stress. These results are consistent with those of other studies that also showed a significant reduction in salivary cortisol levels in patients who received music therapy [54,55]. On the other hand, as for the possible relation of the acute stress level improvements with emotions, a hyperactive HPA axis, characterized by high cortisol levels, is directly associated with the presence of negative emotions [88]. In this way, saliva cortisol levels are positively related to the perception of sadness [89], fear [90], or anger [91] which are, precisely, the variables that improved only in the group that received the intervention and that showed a significant decrease in saliva cortisol.

Furthermore, as for the protocol that was followed, if we compare this study with previous ones that used group adaptations of BMGIM [68,69], the structure of the sessions was practically identical. As for the frequency of treatment, our study was also very similar to these previous studies (one time per week, 2 h each session). With regard to the total duration of the treatment, there were some differences, since our study was carried out in eight sessions, which was slightly below the durations of the other studies indicated. In this regard, the study by Bonde [61] had 10 sessions, and the study by Torres, Pedersen, and Pérez-Fernández [60] was performed with a cycle of 12 sessions. As for the results obtained in our study, they are not comparable as they involve very different populations, although they all refer to a benefit or improvement in patients.

To our knowledge, this is the first study to address both these issues in IBD. It is for this reason that the results obtained in this study, we believe, are relevant and, in our view, they should be further explored (especially in terms of maintaining the effect over time after stopping therapy). Nevertheless, this study presents some limitations. First, the results should be interpreted with caution when attempting to apply them more generally, since the sample was small, non-probabilistic, and restricted to patients living in the Region of Valencia. Future studies must consider a larger sample, in order to be able to make broader comparisons. Second, a large number of patients refused to participate, owing mainly to timetable incompatibility. Nevertheless, the results obtained seem to make an important contribution to the field. Thirdly, there were basal differences between the groups (*p* < 0.05) in some variables, such as patients not taking any type of treatment and patients taking anti-TNF, which could have influenced the study results, especially in the levels of the biochemical markers IgA or cortisol. Furthermore, the possible influence of the phase of the disease should be outlined, i.e., being in a period of remission. Possibly, the impact was not as important as that which would be expected for patients with the disease in an active phase. Therefore, it would be necessary to replicate this study with patients an active phase of the disease in the future. Finally, throughout the duration of the intervention, subclinical relapses of not diagnosed intestinal disease or concomitant diseases could not be ruled out and may have also influenced IgA and cortisol levels during the study period.

As indicated by the World Health Organization (WHO), health is the interaction of multiple social, political, economic, cultural, and scientific factors, thus making this concept a complex reality that must be addressed using an interdisciplinary approach [92]. Therefore, it is especially important to establish research lines aimed at detecting the demands of health professionals and patients and their families as a whole in order to meet their needs and promote quality care in the health system. For these reasons, this research will help optimize the development of specialized interventions that seek to improve the health and quality of life of patients and their families.

## Figures and Tables

**Table 1:** Demographic data and baseline clinical characteristics.

	Intervention Group N = 22	Control Group N = 21
	Median (range)	Median (range)
Age, years	50.5 (28–69)	46.0 (20–60)
Activity index		
UC = Mayo	1.00 (0–2)	2.00 (1–2)
CD = Harvey	2.50 (0–4)	2.00 (1–4)
Time with IBD, years	8.0 (2–42)	9.0 (3–35)
	n (%)	n (%)
Sex	9 M (40.9%)/13 W (59.1%)	10 M (47.62%)/11 W (52.38%)
IBD type		
UC	10 (45.45%)	10 (47.62%)
CD	12 (54.54%)	11 (52.38%)
Montreal UC Montreal CD	E1 = 3 (30%); E2 = 3 (30%); E3 = 4 (40%) A1 = 1 (8.33%); A2 = 8 (66.66%); A3 = 3 (25%); B1 = 7 (58.33%); B2 = 4 (33.33%); B3 = 1 (8.33%); *p* = 2 (16.66%); L1 = 6 (50%); L2 = 2 (16.66%); L3 = 4 (33.3%)	E1 = 3 (30%); E2 = 3 (30%); E3 = 4 (40%) A1 = 1 (9.09%); A2 = 9 (81.81%); A3 = 1 (9.09%); B1 = 7 (63.63%); B2 = 2 (18.18%); B3 = 2 (18.18); *p* = 2 (18.18%); L1 = 4 (36.36%); L2 = 2 (18.18%); L3 = 5 (45.45%)
Treatment		
No treatment *	3 (13.63%)	0
Mesalazine	13 (59.09%)	6 (28.57%)
AZA/MP/MTX	11 (50%)	10 (47.61%)
Anti-TNF *	5 (22.72%)	7 (33.33%)
Vedolizumab	1 (4.54%)	2 (9.52%)
Previous surgery	5 (22.72%)	3 (14.28%)

UC, ulcerative colitis. CD, Crohn’s disease. IBD, inflammatory bowel disease. AZA, azathioprine. MP, mercaptopurine. MTX, methotrexate. Anti-TNF, anti-tumor necrosis factor alpha. Significant level: * *p* < 0.05.

**Table 2:** Descriptive statistics and results of the ANCOVAs.

		Control Group N = 22				Intervention Group N = 21				ANCOVA		
		Pre-Test		Post-Test		Pre-Test		Post-Test				
Variables	Range	Mean	SD	Mean	SD	Mean	SD	Mean	SD	F(df)	*p*	η^2^
MOOD Questionnaire												
Sadness	0–2	0.65	0.65	0.63	0.54	0.66	0.54	0.30	0.37	8.12 (1.48)	0.01	0.15
Fear	0–2	0.92	0.31	0.88	0.40	0.95	0.43	0.64	0.50	4.29 (1.48)	0.04	0.08
Anger	0–2	0.96	0.40	1.06	0.44	1.01	0.51	0.50	0.43	20.83 (1.48)	0.00	0.30
Happiness	0–2	1.42	0.67	1.38	0.60	1.30	0.60	1.14	0.79	0.76 (1.48)	0.39	0.02
HADS												
Anxiety	0–21	8.94	4.48	7.11	4.13	10.61	4.45	5.81	4.72	1.75 (1.48)	0.19	0.04
Depression	0–21	5.28	4.85	4.83	4.45	5.15	4.28	2.58	3.47	3.94 (1.48)	0.05	0.08
CCVEIIQuestionnaire	9–63	43.89	10.31	43.33	15.87	34.91	9.77	43.13	23.17	2.19 (1.48)	0.15	0.05
Biological markers												
HCC (pg/mg)	-	6.54	4.32	9.90	13.68	8.14	8.19	5.45	6.90	2.07 (1.40)	0.16	0.05
SCC (ηg/mL)	-	9.74	12.17	13.83	13.54	5.34	4.28	2.94	2.76	9.31 (1.36)	0.00	0.22
IgA (mg/dL)	10–30	10.16	7.67	11.64	15.84	5.31	4.96	8.16	8.73	0.20 (1.36)	0.66	0.01

SD, standard deviation; HADS, Hospital Anxiety and Depression Scale; CCVEII, Short-form Questionnaire on Quality of Life in IBD; HCC, hair cortisol concentrations; SCC, salivary cortisol concentrations; IgA, immunoglobulin A.
